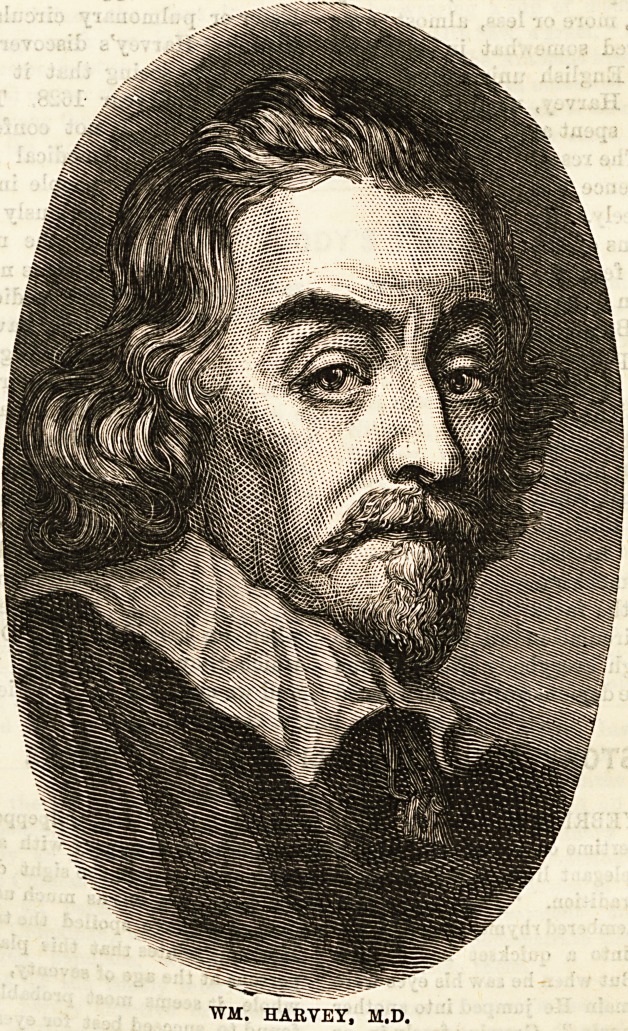# Harvey: An Experimenter

**Published:** 1891-07-18

**Authors:** 


### The Doctor's Armchair.

### HARVEY: AN EXPERIMENTER.

The tepid enthusiasm of the medical profession has proved itself quite unequal to the securing of adequate posthumous honours for some of its most distinguished ornaments. William Harvey, who was the first to clearly demonstrate the circulation of the blood, ought to have been buried in Westminster Abbey at the time of his death. He actually was buried at Hempstead, an unknown Essex village, and there he lay till the vault in which he had been interred fell into ruins. The Royal College of Physicians of London then made a tardy and inconsequent effort to do justice to his memory, and re-interred him, or what remained of him, as the report says, "with befitting solemnity," in the Harvey Chapel attached to the Hempstead Church. Harvey was treated by this learned body after his death in much the same way as he had been treated by the same body during his life. When alive he was allowed to reach the age of seventy-six before he was offered the presidency of his college. The offer came too late, and it was declined. Similarly he was permitted to lie two hundred years in an almost unknown grave before any effort was made to do a little honour even to his bones.

Was Harvey a great man? What constitutes a great man? If we can answer this latter question correctly we may be able to determine whether Harvey was a great man or not. Space does not permit us to make an excursus of that kind. But we may ask—Did Harvey render great and lasting service to the whole of mankind? Most certainly he did. He held up the torch of true medical science and fact in an age of almost Egyptian medical darkness. His discovery and demonstration constituted a beacon light which guided zealous and honest workers to the truly noble medical science under whose beneficent régime we live to-day. If any man wishes to know what are the appropriate honours which should gather about the name of such a discoverer, he has but to duly weigh this fact—that Harvey was one of the most strenuous and enlightened pioneers of a true medical science in times when medicine was little more than an obsolete, misleading, and tyrannical superstition.

WM. HARVEY, M.D.

Certain classes of persons who are much concerned with the medical affairs of the present time will do well to consider some of the facts and circumstances of Harvey's life. Chief among those classes may be mentioned anti-vivisectors, medical educators, and dull-witted professional obstructionists. Let the anti-vivisectors ask themselves two questions: Was it a scientific circumstance of the first importance when the circulation of the blood was demonstrated beyond the possibility of rational doubt? And, secondly, could that demonstration have been convincingly made at all without experiments on living animals? To us of this epoch the circulation of the blood is an affair of the most extreme simplicity. To take the greater circulation as an easy illustration, every educated schoolboy knows that the heart is a central forcing pump; that it discharges its aërated blood into the great arteries; that the great arteries pass it on to the smaller, and that the smaller bring the flow to a kind of ultimatum in the capillaries; the capillaries in their turn gather up the blood and pour it into the smallest veins; the smallest veins into the larger, and the larger into the largest, until finally the stream is returned to the heart again, and after being re-oxidised in the lungs, is once more sent forth by the heart on its errand of nutrition and waste-separation.

All this, we repeat, is child's play now, even to the Board School boy. But when Harvey commenced his investigations there were many men of the highest scientific rank who still believed that the arteries contained air; whilst the very existence of the capillaries, the knowledge of which would have removed at a stroke all difficulties from the way of Harvey's demonstration, was hardly so much as dreamed of. We have the profoundest sympathy with the tender and worthy feelings of anti-vivisectors. If the decision of the question were left with us we would insist that only men of mature age and of proved humanity should be permitted to perform vivisection. But whether the morality of vivisection and vivisectors be high or low—and that is a question we are not now discussing—this much at least is certain, that modern scientific medicine, with all that it includes, is sprung almost entirely from the experimental laboratory.

Harvey, as medical educators will do well to note, did not begin his professional career by the way of apprenticeship to a medical practitioner. He spent six years of his boyhood at the Canterbury Grammar School, and went straight from school to the University. Cambridge was his University and Caius his College. He received, in short, the education of a gentleman. To a medical man, as to others, that is a great advantage. The want of such an education is a deficiency which is almost irreparable. For his medical education Harvey had to go to a foreign university. The English universities at that time despised medicine, and they have continued to despise it, more or less, almost ever since. Medicine has suffered somewhat in England on that account, but the English universities have suffered a good deal more. Harvey, no doubt, gained largely by the five years he spent at the University of Padua. The result of his long academic experience was that he learnt to think freely. He did not accept the traditions of his time as final; he did not fear the great names which were then famous in the medical profession. Before he was forty years of age he expounded, as lecturer to the College of Physicians, his reasoned convictions, not only that the arteries did not contain air, but that they did contain blood.

It is amusing to read that when Harvey accompanied the Earl of Arundel on his embassy to the Emperor, he publicly demonstrated to Caspar Hofmann the facts which made the arterial circulation an indisputable necessity; and that although everybody else who was present was convinced by the demonstration, the learned Professor, who had long been one of the sturdiest opponents of the new theory, refused to budge so much as a single inch from the orthodox position. The dull-witted among doctors, of whom there are not a few, should take this lesson to heart. Hofmann no doubt thought himself a very superior person to Harvey; as a matter of fact he was simply a dull and obstinate fool.

It is only fair to add that Galen, who lived and wrote fourteen hundred years before Harvey, disputed the prevalent view that the arteries contained air; and it may be taken for granted that this opinion of Galen received consideration at different periods from medical men of original mind in all countries. To Servetus, the liberal-minded opponent of Calvin's sour theology, the lesser or pulmonary circulation owed its first demonstration. Harvey's discovery is not yet three hundred years old, seeing that it was first published to the world in the year 1628. That is a fact which does not confer much honour on the medical men of past times. Some people indeed seem to think that it seriously detracts from the greatness of the medical science of to-day That is nonsense. On the contrary, the medical profession of the present time, having such a comparatively undistinguished professional ancestry, deserves all the more honour, in that it has to so large an extent cast aside its ignorant superstitions, and now stands before the world as the generous advocate of scientific freedom in every form. That medical men would be none the worse if they were denuded of a few more of their prejudices we do not deny. But when we consider what David calls the "pit from which they were digged," we cannot but be grateful, and we must try to be patient.

## Figures and Tables

**Figure f1:**